# Tracing the composition of single e-cigarette aerosol droplets in situ by laser-trapping and Raman scattering

**DOI:** 10.1038/s41598-020-64886-5

**Published:** 2020-05-13

**Authors:** Grégory David, Evelyne A. Parmentier, Irene Taurino, Ruth Signorell

**Affiliations:** 1 Department of Chemistry and Applied Biosciences, ETH Zurich, Vladimir-Prelog-Weg 2, CH-8093 Zurich, Switzerland; 2 PMI R&D, Philip Morris Products S.A., Quai Jeanrenaud 5, CH-2000 Neuchâtel, Switzerland

**Keywords:** Physical chemistry, Chemical physics, Risk factors

## Abstract

The partitioning of components between droplets and the gas phase in e-cigarette aerosols has a significant impact on deposition within the respiratory tract. However, exclusive detection of droplet composition has, so far, been elusive. Consequently, the dynamics of partitioning between droplets and the gas phase remains unknown. Here, we combine optical trapping of single droplets with *in situ* Raman scattering for destruction-free monitoring of e-cigarette droplet composition with a time resolution of seconds. We find that the initial droplet composition is very close to the composition of the e-liquid. Upon dilution with air, the droplet composition changes exponentially on a time scale of seconds, mainly because of evaporation of propylene glycol. The nicotine content in the droplet is controlled by the pH. Nicotine evaporates from the droplets under basic conditions, but remains in the liquid under acidic conditions. These results are crucial for advancing e-liquid research and manufacturing.

## Introduction

Electronic cigarettes (e-cigarettes) represent a multibillion dollar industry^1^ and a fast growing market^2^. The on-going debates on e-cigarettes have made it very clear that more studies are needed^3,4^ for better assessing their health effects and potential to reduce the risk of smoking-related diseases as compared to continued smoking^5–8^ and, eventually, for designing products^4^. E-cigarettes are devices designed to deliver nicotine to the user through aerosol droplets generated by heating up a liquid called “e-liquid”. Most e-liquids are composed of propylene glycol (PG), vegetable glycerol (VG), nicotine, flavoring supplements, and water^9,10^. Heating such an e-liquid evaporates all of its chemical compounds into the gas phase. When the gas phase cools down, liquid aerosol droplets are formed by condensation of the vapor. The different components of the e-liquid are then present both in the droplet and gas phases of the aerosol. Many studies have underlined the importance of and need for knowing which components remain in the droplet phase and which evaporate again into the gas phase^11–14^. The partitioning of components between the droplet and gas phases, for example, affects their deposition in the respiratory tract^11–14^.

Yet, the dynamics of compound transfer between the droplet and gas phases remains largely unknown, mainly because of the lack of adequate *in situ* methods for exclusively probing the composition of the droplets in the aerosol with adequate time resolution. A few simulations have addressed this topic^11,13^, but, to the best of our knowledge, no corresponding experimental data are available. Many experimental methods measure the chemical composition of the whole e-cigarette aerosol, i. e. without distinction between the droplet and gas phases and without time resolution. Most measurements are off-line measurements that first sample the e-cigarette aerosol and then use *ex situ* analysis methods, typically chromatography and/or mass spectroscopy^1,14,15^. These off-line measurements usually sample both the gas and droplet phases^1,14,15^, but new filters are being developed in an attempt to separate the droplet phase from the gas phase before analysis^16^. However, considering the fairly high volatility of several of the e-cigarette components, combined with the lack of time resolution, the use of filters does not seem to be the most appropriate method. Generally, sampling and extraction can strongly affect the chemical composition of both the droplets and the gas phase^17–20^, and, because of the lack of adequate time resolution, they cannot provide real-time compositions.

A couple of real-time measurements of the composition of e-cigarette aerosols have been reported^19,21^. Because they are not affected by deposition or extraction, such real-time data are likely more representative of the actual aerosol composition than data retrieved by off-line methods. Nevertheless, real-time measurements are performed either on the whole aerosol (droplets and gas phase together)^19^ or on the gas phase only^21^. Information exclusively on droplet composition cannot be retrieved from these studies. Furthermore, the air sampling required for these real-time measurements might affect the e-cigarette droplets, especially if a high air dilution is required^22^.

This study reports the first real-time measurements of the composition of single e-cigarette droplets, performed *in situ* in the aerosol phase (i.e., without sample extraction) and with a time resolution of seconds. To this end, we isolated single droplets directly in the e-cigarette aerosol by using an optical trap and recorded the temporal change in the droplet composition *in situ* by means of Raman scattering. Optical trapping allowed us to isolate single particles in air for an extended period of time^23–26^ and thus monitor time-dependent processes affecting the particles, such as evaporation^25,27^, diffusion^28^, chemical reaction^29,30^, and phase transitions^31^ or phase separations^32,33^. We report changes in chemical composition on the second timescale for droplets generated from four different e-liquids with the same ratio of PG and VG but with different nicotine concentrations and pHs.

## Results

### Time-resolved single-droplet Raman scattering

**Figure 1:**
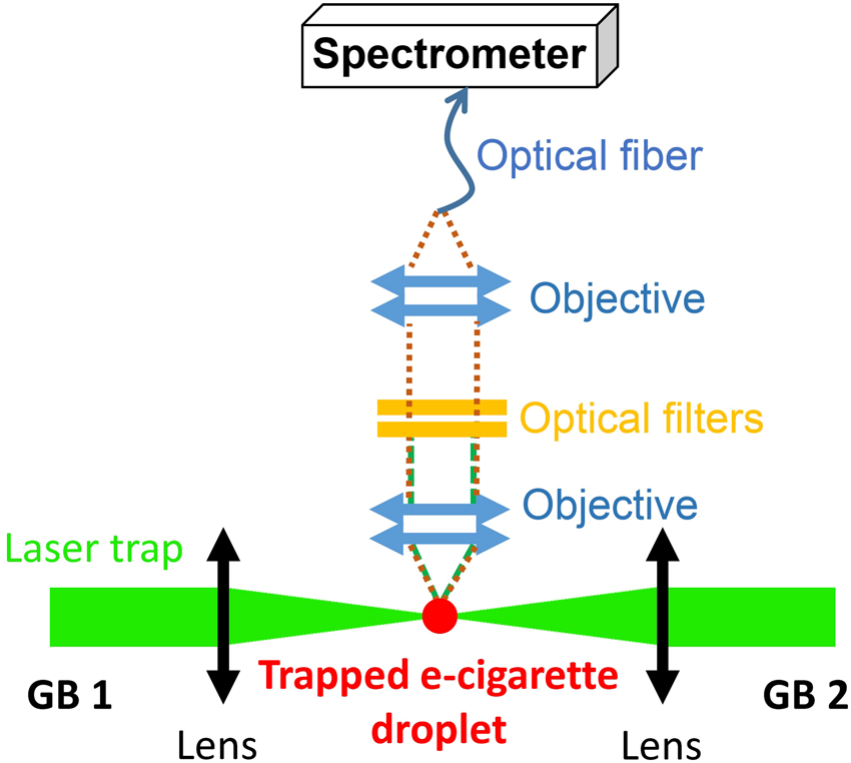
Sketch of the experimental setup. It consists of an optical trap (counter-propagating optical tweezers; CPT) and a Raman spectrometer. The CPT trap is formed by two focused counter-propagating Gaussian beams (GB 1 and GB 2). The e-cigarette aerosol droplet is trapped in the common focus of GB 1 and GB 2. The Raman scattering of the droplet is collected and filtered by the collection optics (objectives and optical filters) and detected in a fiber-coupled spectrometer.

The experimental setup couples counter-propagating optical tweezers^34^ (CPT) with single-droplet Raman scattering measurement^31^ (Fig. 1; see Methods). The CPT isolates a single droplet in air from a droplet plume generated by the e-cigarette. The plume is subsequently diluted by a humidified air flow (20 °C and 65% relative humidity (RH)). Raman scattering is used to monitor the concentration of the e-cigarette compounds (PG, VG, nicotine, and water) in the droplet with a typical time resolution of ~3 s. The single-droplet Raman setup allows us to exclude practically all contributions from the gas phase and record the chemical composition of the droplet itself. All concentrations are given as mass percent (%mass) of the respective compounds in the aerosol droplet or in the e-liquid used in the e-cigarette. Typical standard deviations of the droplet concentrations are on the order of 0.3%. Bulk solutions of different concentrations are used for calibration (Supplementary Information). Aerosol droplets are formed from four e-liquids with the same VG–PG ratio (%VG/(%VG + %PG) = 0.3; Supplementary Information) and the following nicotine concentrations and pH: 2% nicotine at pH 9.9; 5% nicotine at pH 9.9; 5% nicotine at pH 6.5; and 5% nicotine at pH 3.4.

### Time- and pH-dependence of the nicotine concentration in the droplets

**Figure 2:**
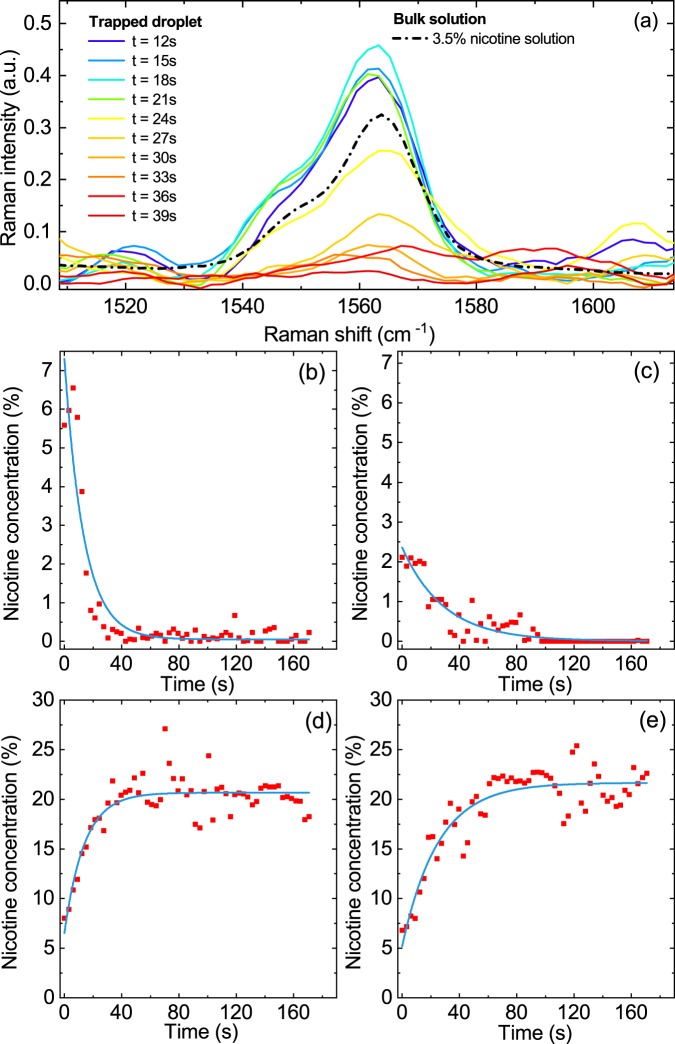
Time-dependence of the nicotine content in e-cigarette aerosol droplets. **(a)** Typical time evolution of the nicotine Raman band recorded for a droplet generated from an e-liquid with 5% nicotine and pH 9.9 (colored lines). The dashed-dotted black line is a Raman spectrum of a bulk liquid with 3.5% nicotine. **(b–e)** Time-dependence of the nicotine concentration in droplets generated from different e-liquids: **(b)** 5% nicotine at pH 9.9; **(c)** 2% nicotine at pH 9.9; **(d)** 5% nicotine at pH 6.5; and **(e)** 5% nicotine at pH 3.4. The light blue lines represent exponential fits to the time evolution of the experimental nicotine concentration (Methods and Table 1).

Figure 2a shows the temporal evolution (full, colored lines) of the Raman band of nicotine at ~1565 cm^−1^ of a single trapped droplet that was generated from an e-liquid with 5% nicotine and pH 9.9. The dashed-dotted black line shows a liquid bulk spectrum with 3.5% nicotine for comparison (Supplementary Information). The fast decrease in the intensity of the droplet Raman band results from the evaporation of dissolved nicotine. After about 30 s, the nicotine has completely disappeared from the droplet. The corresponding time evolution of the nicotine concentration is presented in Fig. 2b. The nicotine concentration in the droplet decreases with a time constant of τ = +9.4 ± 3.7 s (Table 1 and Methods) until it reaches the detection limit. A similarly fast conversion of dissolved nicotine to gaseous nicotine with a time constant of τ = +21.7 ± 8.1 s is also observed for droplets generated from an e-liquid with a lower nicotine concentration of 2% at the same pH (pH 9.9; Fig. 2c; Table 1).

**Table 1:** Characteristic time τ of the decrease (+sign) or increase (−sign) in the concentration of nicotine (nic), VG, PG, and water (H_2_O) in e-cigarette aerosol droplets generated from four different e-liquids. Reported are average values and standard deviations of τ retrieved from measurements of 5 or 6 single droplets generated from the same e-liquid.

E-liquid properties	τ^nic^ (s)	τ^VG^ (s)	τ^PG^ (s)	τ^H2O^ (s)
5% nicotine, pH 9.9	+9.4 ± 3.7	−7.4 ± 4.4	+12.0 ± 5.9	−7.0 ± 5.2
2% nicotine, pH 9.9	+21.7 ± 8.1	−9.74 ± 3.7	+8.8 ± 3.6	−12.7 ± 4.3
5% nicotine, pH 6.5	−11.0 ± 5.5	−18.8 ± 4.7	+16.2 ± 7.0	*
5% nicotine, pH 3.4	−31.2 ± 20.5	−24.7 ± 17.4	+28.2 ± 25.1	*

*These values could not be retrieved because the variation of the pH during droplet evaporation is unknown, and the retrieval of the H_2_O concentration in the droplet from the OH Raman band depends on the pH of the droplet.

This behavior is completely reversed at a lower pH (pH 6.5 and 3.4; Fig. 2d,e, respectively). Instead of a decrease, we now observe an increase in nicotine concentration in the droplets over time. At both pH 6.5 and pH 3.4, the initial nicotine concentration of ~5% rapidly increases (with time constants of τ = −11.0 ± 5.5 s and τ = −31.2 ± 20.5 s, respectively; Table 1) until it stabilizes at about 20% after ~40 s. A low pH drastically reduces the volatility of nicotine, so that its concentration increases as PG evaporates from the droplet. With VG being much less volatile than any of the other components (nicotine, PG, and H_2_O), its evaporation from the droplet is assumed to be negligible (see below). These results demonstrate how the pH of the e-liquid dictates the time-dependence of the nicotine concentration in the droplets and, hence, its partitioning between the liquid and gas phases.

Time-resolved single-droplet Raman spectroscopy also allows us to compare the initial droplet concentrations (recorded directly after aerosol formation) with the concentrations of the original e-liquid. Figure 2b–d show that the initial nicotine concentration in the droplets is fairly close to the nicotine concentration of the corresponding e-liquid from which the droplet was generated, with a tendency to somewhat higher initial nicotine concentrations. A similarly close agreement is found between the initial VG–PG ratio in the droplets and that in the e-liquid from which the aerosol was formed (see below).

### Time-dependent concentrations of VG, PG, and H_2_O in the droplets

**Figure 3:**
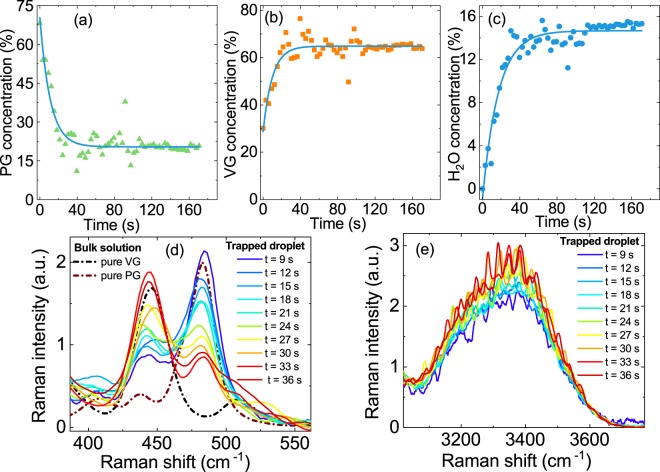
Time-dependence of the PG, VG, and H_2_O content in e-cigarette aerosol droplets. Time-dependence of the concentration of **(a)** PG, **(b)** VG, and **(c)** H_2_O for an example of a droplet generated from an e-liquid with 2% nicotine and pH 9.9. Light blue lines: Exponential fits to the experimental concentrations (Methods and Table 1).Time-evolution of the corresponding Raman spectra in the region of the **(d)** VG and PG band and **(e)** OH-stretch band. Measurements for pure VG and pure PG bulk solutions are shown as references.

Figure 3 summarizes the typical time evolution of the concentrations of VG, PG, and H_2_O in the droplets for the example of an e-liquid with a nicotine concentration of 2% and pH 9.9. The initial PG concentration of ~68% in the droplet lies close to the e-liquid PG concentration of 69% (Fig. 3a). Because of evaporation of PG from the droplet, a pronounced decrease in PG concentration of more than a factor of three is observed within the next ~20 s, before the PG concentration stabilizes at ~20%. The characteristic times τ for the decrease in PG concentration for droplets formed from the different e-liquids are provided in Table 1. This type of kinetics with stabilization of the PG concentration at a finite value indicates pronounced intermolecular interactions between PG and the other compounds (mainly VG) in the droplet. To support this explanation, we calculated the evaporation kinetics of PG in droplets that consist of 80% PG and 20% VG, neglecting any intermolecular interactions between PG and VG (see equations in ref. ^35^). For a model droplet of 5-μm radius, these calculations predict complete evaporation of PG within only a few seconds (even faster if the particle is smaller). Comparison with the experimental results thus reveals that the presence of VG decreases the activity coefficient of PG (as explained in Supplementary Information). The observed increase in VG concentration from ~30% to 65% within the first 30 s (Fig. 3b) is mainly due to the evaporation of PG over this time span. There is no VG source in the system that could lead to an increase in the VG concentration by condensation, and the contribution of the evaporation of nicotine (only relevant at pH 9.9) is smaller than that of the evaporation of PG. The concentration of H_2_O increases by approximately one order of magnitude from ~0% to 16% (Fig. 3c), with a similar time-dependence as that observed for VG. Condensing water from the humidified air is the main source that determines the water content in the droplet. The characteristic times τ for the increase in VG and H_2_O concentrations are provided in Table 1. For all four e-liquids, we find similar trends in the time-dependence of all droplet components.

Figure 3 presents also the time-evolution of the droplet Raman spectra in the region of the characteristic bands of VG and PG between 430 and 500 cm^−1^ (panel d) and in the region of the broad H_2_O stretching band around 3300 cm^−1^ (panel e). The mass concentrations of PG, VG, and H_2_O in panels a to c are retrieved from these Raman spectra by using the calibration described in the Supplementary Information. The droplet spectra show an increase in the intensities of the VG (450 cm^−1^) and water (3300 cm^−1^) bands, which is accompanied by a decrease in the PG band intensity (480 cm^−1^) over time.

### Mass changes of e-cigarette aerosol droplets

**Figure 4:**
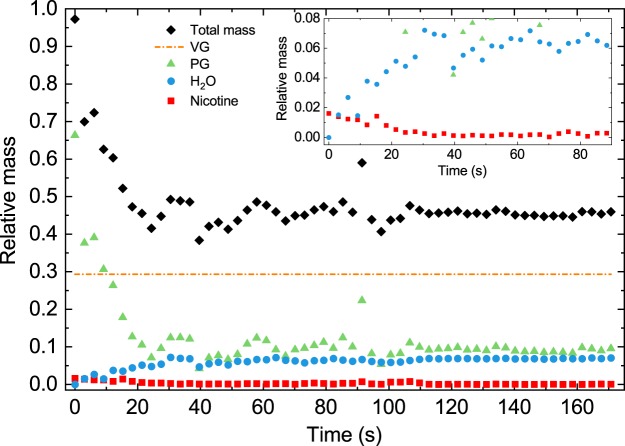
Temporal mass changes of an e-cigarette aerosol droplet. Total mass change of a droplet (black diamonds) over time relative to the initial droplet mass (2% nicotine at pH 9.9). Mass changes of the different components over time, relative to the initial mass: VG (orange, dash dotted line), PG (green triangles), nicotine (red squares), and H_2_O (blue dots). The VG mass is assumed to be constant. The inset shows the nicotine and H_2_O mass changes in more detail.

The data in Fig. 3 can be used to calculate the total mass change of the droplet phase and the mass changes of the different compounds over time, relative to the initial mass. The mass of VG is assumed to stay constant over time. Its mass is used as the reference mass to calculate the mass changes of all other components. The assumption of an approximately constant VG mass is supported by the low volatility of VG (vapor pressure, 11.6 mPa^36^). A pure VG droplet of 4-μm radius, for example, would only lose 2% of its initial mass within 40 s. In the mixture, the evaporation rate of VG is likely to be even lower. Figure 4 shows that, within ~15 s, more than 50% of the total droplet mass evaporates into the gas phase (black diamonds), which is mainly because of PG evaporation (green triangles). Less than 15% of the initial PG mass remains in the droplet. Furthermore, the inset in Fig. 4 shows that, in an e-liquid with 2% nicotine and pH 9.9, nicotine disappears from the droplet (red squares), while the water mass in the droplet increases pronouncedly (blue dots). Figure 4 illustrates again that the droplet composition stabilizes after about 20 s and remains constant for at least 170 s.

## Discussion

The different compounds in e-cigarette aerosols can be present in both the droplet and gas phases. The need to understand the partitioning and its temporal evolution between the two phases has been underlined many times, but has so far been elusive. This study presents the first *in situ* measurements of the composition of single e-cigarette aerosol droplets generated from e-liquids with different nicotine concentrations and pHs. The time evolution of nicotine concentration in the droplets upon dilution with humidified air is dictated by the pH of the e-liquid (Fig. 2).

Under basic conditions (pH > pK_A,2_, with pK_A,2_ = 7.9), nicotine is mostly present in its neutral form, which completely evaporates from the droplet within a few ten seconds. When the pH lies in the slightly acidic region (pK_A,1_ < pH <pK_A,2_, with pK_A,1_ = 3.2), nicotine is present in a charged (singly or doubly protonated) state. This form is nonvolatile and stays in the droplet. Because PG is still evaporating, decreasing in concentration from ~68% to ~20% within a few ten seconds, the mass concentration of nicotine in acidic droplets necessarily increases from initially a few percent to 20% on the same time scale. This effect can be exploited to control the partitioning of nicotine between the droplet and gas phases through the pH of the liquid. In every case, the observed droplet composition reaches a stationary state on the same time scale of a few ten seconds (Figs. 2 and 3). The PG mass concentration has decreased to a point where the rate of evaporation becomes so small that the composition of the droplet does not change significantly within the time window of our observation (~170 s). During the whole process, the H_2_O content of the droplet is constrained by the relative humidity (RH). The change in the total mass of the e-cigarette droplet during evaporation into the gas phase within the first few ten seconds is substantial. Typical total mass changes are on the order of a factor of two (Fig. 4), mainly because of evaporation of PG. More than 85% of the initial PG mass in the droplet phase evaporates into the gas phase. Under basic conditions, most of the initial nicotine mass in the droplet (more than 95%) is converted into gaseous nicotine, while, under slightly acidic conditions, all nicotine that initially condenses into the droplets remains preserved in the droplets. These measurements were performed at 65% RH and 20 °C. At higher RH (expected in the human body), the uptake of water by the droplets will increase, while the behavior of VG, PG and nicotine in the droplets is not expected to change significantly. At higher temperature, the evaporation kinetics are expected to be enhanced because the vapor pressure of the compounds will increase.

We followed the approach of detecting the composition of e-cigarette droplets *in situ* rather than detecting only the gas phase or both the droplet and gas phases together. In contrast to conventional methods, our laser-trapping-Raman scattering approach allows us to trace the temporal evolution of the droplet composition without destroying the droplets and without contributions from the gas phase. Our study demonstrates that the chemical composition and total mass of e-cigarette droplets undergo large changes on a time scale of a few to a few ten seconds upon dilution of the aerosol plume with humidified air. Destruction-free single-droplet techniques that allow us to monitor the partitioning of compounds between the droplet and gas phases *in situ* open up new avenues for future research on e-cigarette droplets and for e-liquid manufacturing. Tracing droplet composition and evaporation of compounds into the gas phase will also be very valuable for assessing the deposition of e-cigarettes emissions in the respiratory track and thus for better understanding their health impact.

## Methods

### Generation of e-cigarette aerosol droplets and environmental control

The e-cigarette (Nicocig MESH) generates a droplet plume by complete evaporation of the e-cigarette liquid and subsequent condensation. Part of the plume of a 30-mL puff is delivered to the trapping cell with a programmable dual syringe pump over a duration of 1.95 s using a boxcar profile. Single e-cigarette droplets are isolated in the trapping cell by CPT. The trapping cell allows us to control the environment of the trapped particle by exposing it to a humidified air flow of 65% RH, which simulates dilution of the original plume by mixing with additional wet air of the same RH and temperature. Droplet sizes vary by up to several microns. A more precise quantification of the droplets size was not possible with the techniques used for the reported measurements.

### Optical trap

A detailed description of the CPT optical trap is provided in previous reports^31,34^ (Fig. 1). A 532-nm continuous-wave laser (Opus 532, Laser Quantum; typical power 5 W) is first expanded fourfold with a two-lens telescope. The beam is then split into two counter-propagating beams (GB1 and GB2) with similar power by coupling a half-wave plate (λ/2) and a polarization beamsplitter cube. Finally, a 56.6-mm focal length lens is used to focus each beam in the center of a trapping cell.

### Raman scattering

The experimental setup is as described previously^31^ and shown in Fig. 1. The light scattered by the droplet (elastic and inelastic Raman scattering) is collected with a microscope objective (20x; 0.42 numerical aperture; 20 mm working distance). Notch filters are used to eliminate the elastically scattered light. A second objective is used to couple the remaining inelastically scattered light to the fiber of the spectrometer (Kymera spectrometer with a Newton DU940P-BV camera, Andor). The Raman spectra of the trapped e-cigarette droplets are recorded with a few seconds integration time (typically 3 s). As shown in the Results section, the chemical composition of the e-cigarette droplets changes on a time scale of seconds.

The same collection optics and spectrometer are used to measure the Raman scattering of bulk liquids of known chemical composition (Supplementary Information). These bulk spectra are used to generate calibration curves for determining the time-dependent composition of the e-cigarette droplets. To optimize the signal-to-noise ratio, liquid bulk spectra are accumulated over a few hundred seconds.

### Time-dependence of droplet composition

The time-evolution of the concentrations of nicotine, VG, PG, and H_2_O are described by a characteristic time τ, which is retrieved from the fit of Eq. (1) to the experimentally observed concentration of the respective compound (Figs. 2 and 3):

1 $$C(t)={C}_{0}+A\cdot \exp (\frac{A}{|A|}\frac{t}{\tau })$$

C(t) is the concentration of the compound considered at time t. C_0_, A, and τ are the fit parameters. The factor $\frac{A}{|A|}$ in the exponential is introduced to obtain a different sign of τ for decreasing (A < 0, τ > 0) vs. increasing (A > 0, τ < 0) concentrations.

## Supplementary information

Supplementary information.
