# Supplementary material for: Tracing the composition of single e-cigarette aerosol droplets in situ by laser-trapping and Raman scattering
Source: Sci Rep. 2020 May 13;10:7929. doi: 10.1038/s41598-020-64886-5 (PMC7220912; doi:10.1038/s41598-020-64886-5)
Supplement: Supplementary file 1 — Supplementary information. [file 41598_2020_64886_MOESM1_ESM.docx]

# Supplementary information

**Tracing the composition of single e-cigarette aerosol droplets in situ by laser-trapping and Raman scattering**

Grégory David, Evelyne A. Parmentier, Irene Taurino, and Ruth Signorell*

# Raman measurements of bulk solutions and calibration

The same collection optics and spectrometer as those for single e-cigarette droplets (see Methods) are used to record Raman scattering from bulk solutions. Supplementary Fig. 1 shows a scheme of the experimental setup for bulk measurements. The calibration curves for determining the composition of the trapped e-cigarette droplets are retrieved from the Raman spectra of bulk solutions of known chemical composition. The calibration curves for the different compounds are presented hereafter. They all use the area of a characteristic Raman band to retrieve the concentration of the corresponding compound. To compare the area of a characteristic Raman band among different spectra, all Raman spectra are first normalized by their total integral determined in the region from 200 to 3000 cm^-1^.


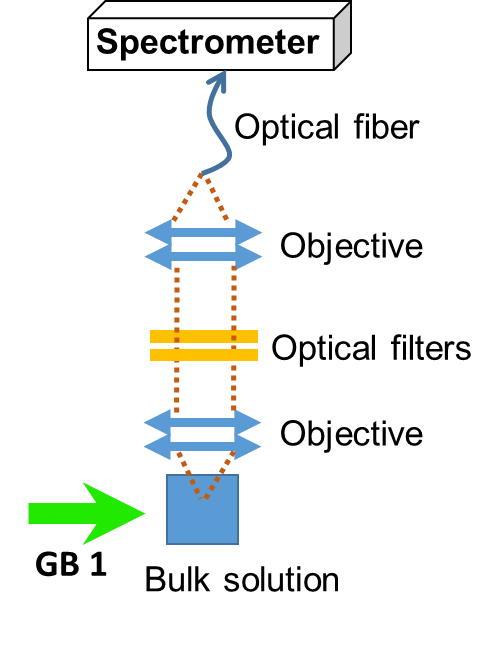


**Supplementary Fig. 1**. Optical setup used to collect and detect the Raman scattering of bulk solutions.

# Definition of concentrations and VG–PG ratio

All concentrations are presented as mass concentration expressed in percentage (%mass) of the total droplet mass or the total mass of the bulk solution.

As a simple indicator of the relative concentrations of VG and PG in the droplets or bulk solutions, we define the VG–PG ratio as %VG / (%VG + %PG).

# Calibration of nicotine concentration

The calibration of nicotine concentration is shown in Supplementary Fig. 2a for bulk solutions with different VG–PG ratios (from 0.3 to 1) and nicotine concentrations. The nicotine band at ~1570 cm^-1^ does not overlap with any bands of other compounds and is thus used for nicotine calibration. Because the area under the nicotine band turns out not to depend on the VG–PG ratio of the solution, measurements with different VG–PG ratios are fitted together. The calibration curve thus obtained for the nicotine concentration is linear (panel (b)).


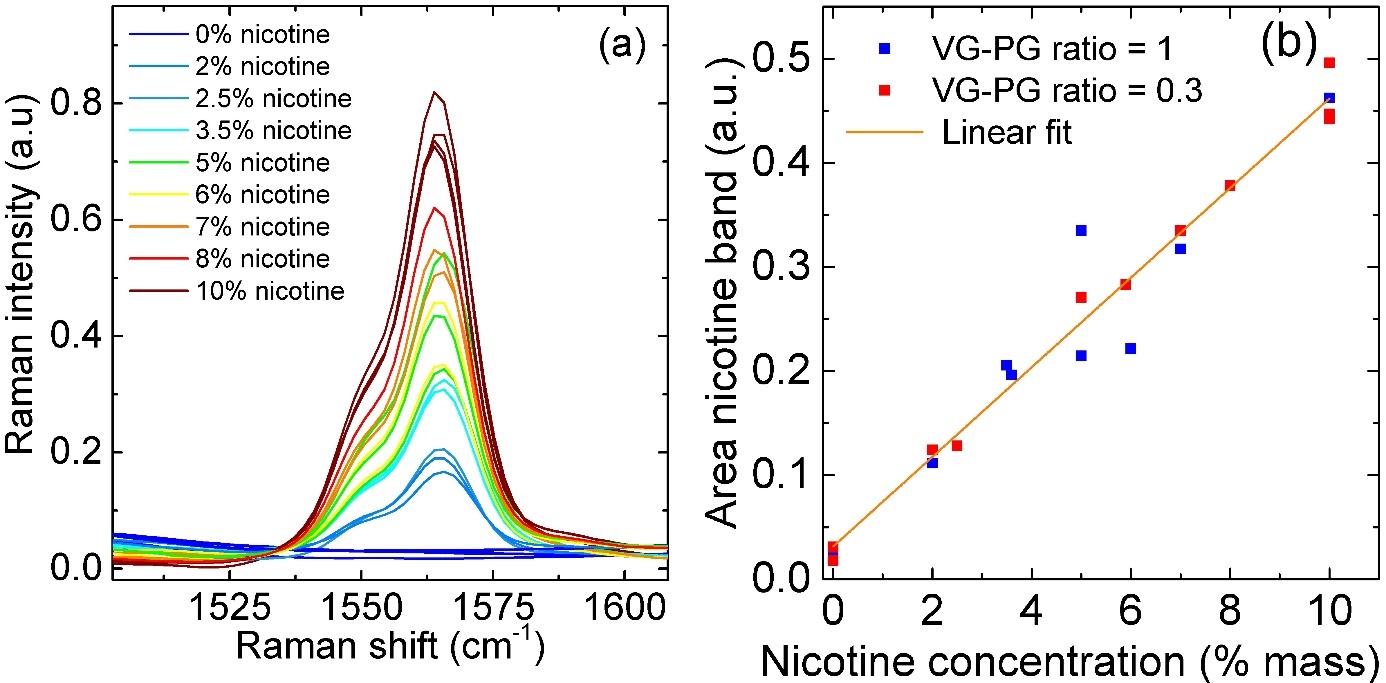


**Supplementary Fig. 2. Calibration of nicotine**. **(a)** Raman spectra in the region of the nicotine band around 1570 cm^-1^ for bulk solutions with different nicotine concentrations. **(b)** Calibration curve for nicotine. The spread of some data points in the calibration likely arises from inhomogeneities in the bulk solutions due to the high viscosity of VG.

# Calibration of the VG–PG ratio

The Raman spectra in the region of the VG and PG bands for bulk solutions with VG–PG ratios between 0.3 and 1 are shown in Supplementary Fig. 3a. This range of VG–PG ratios is chosen to cover the whole range of concentrations of the e-cigarette droplets (see Results). The ratio of the areas under the VG band around ~450 cm^-1^ (highlighted in grey) and PG band around ~490 cm^-1^ (highlighted in orange) yield the calibration curve in panel (b). From the VG–PG ratio (*Rat_VG–PG_*), the concentrations of VG and PG ([VG] and [PG] respectively) are obtained under the assumption [VG] + [PG] + [H_2_O] + [nicotine] = 1:

$$[VG]=Rat_{VG-PG}\left(1-[nic]-[water] \right)$$

$$[PG]=\left(1-Rat_{VG-PG}\right)\left(1-[nic]-[water] \right)$$

where [H_2_O] is the water concentration and [nicotine] is the nicotine concentration.


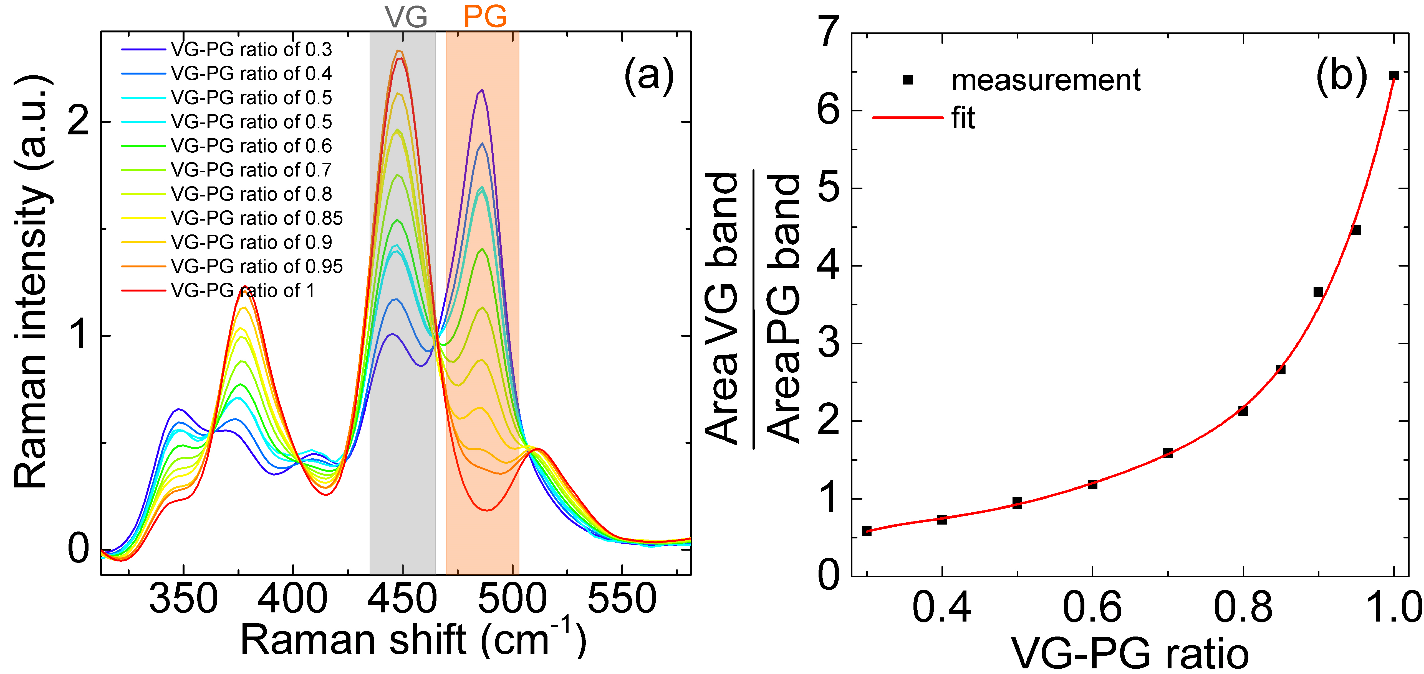


**Supplementary Fig. 3. Calibration of the VG–PG ratio.** **(a)** Raman spectra in the region of the VG band at ~450 cm^-1^ (highlighted in grey) and PG band at ~490 cm^-1^ (highlighted in orange) for bulk solutions with different VG–PG ratios. **(b)** Calibration curve for the VG–PG ratio.

# Calibration of the water concentration

Supplementary Fig. 4a shows the Raman spectra of bulk solutions with different water concentrations and VG–PG ratios. The band that changes the most with water concentration is the OH-stretch band between 3000 and 3600 cm^-1^. Note that the area of this band depends both on the water concentration and VG–PG ratio. Panel (b) quantifies the area of the OH-stretch band as a function of the water concentration for three different VG–PG ratios (0.3, 0.65, and 1). Panel (c) shows that the area of the OH-stretch band at constant water concentrations depends linearly on the VG–PG ratio. The corresponding parameters of the linear fits are listed in Supplementary Table 1. Hence, for all VG–PG ratios, the appropriate calibration curve for water concentration can be determined from linear interpolations between the curves shown in panel (b).


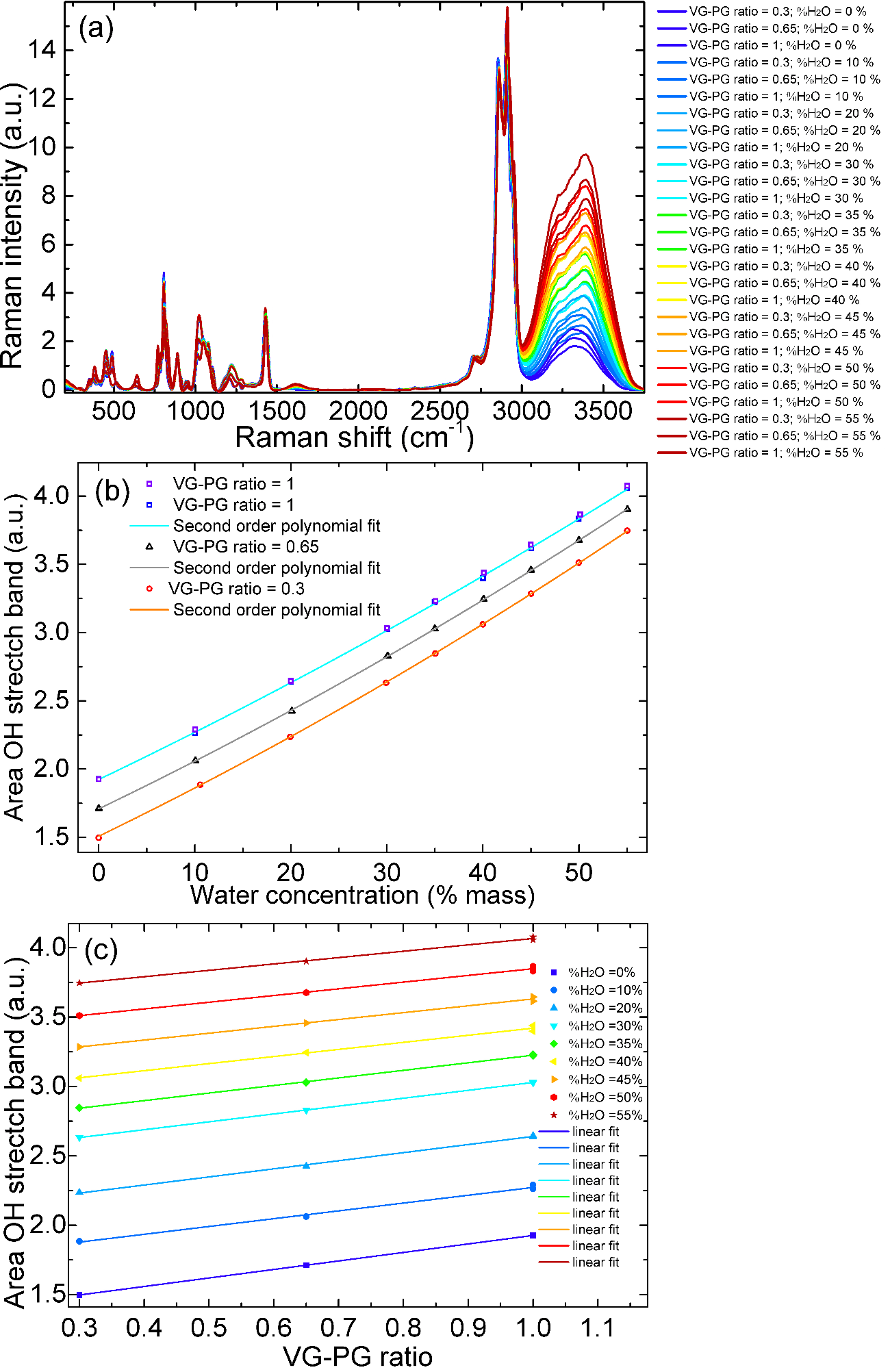


**Supplementary Fig. 4. Calibration of water content.** **(a)** Raman spectra for bulk solutions with different water concentrations and VG–PG ratios. The color code of the lines indicates the water concentration of the solution with increasing water content from blue to red. **(b)** Area of the OH-stretch band as a function of the water concentration for three different VG–PG ratios. **c)** Area of the OH-stretch band as a function of the VG–PG ratio for 9 different constant water concentrations.

**Supplementary Table 1. Parameters of the linear fits shown in panel (c) of Supplementary Fig. 4.**

| Water concentration (%) | Intercept | Intercept standard error | Slope | Slope standard error |
| --- | --- | --- | --- | --- |
| 0 | 1.3129 | 4.87E-04 | 0.00613 | 6.15E-06 |
| 10 | 1.7091 | 0.0240 | 0.00563 | 3.02E-04 |
| 20 | 2.0560 | 0.0125 | 0.00584 | 1.57E-04 |
| 30 | 2.4613 | 0.0038 | 0.00567 | 4.82E-05 |
| 35 | 2.6795 | 0.0085 | 0.00545 | 1.08E-04 |
| 40 | 2.9091 | 0.0285 | 0.00511 | 3.59E-04 |
| 45 | 3.1365 | 0.0205 | 0.00494 | 2.59E-04 |
| 50 | 3.3651 | 0.0219 | 0.00483 | 2.76E-04 |
| 55 | 3.6070 | 0.0143 | 0.00459 | 1.80E-04 |

# Identification of compound responsible for the activity coefficient change of PG.

We have calculated the evaporation kinetics of PG in droplets that consist of 80% PG and 20% VG, neglecting any intermolecular interactions between PG and VG (ideal mixture, i. e. activity coefficients 1). In other words, we used the vapor pressure of pure PG and pure VG to simulate how fast all the PG evaporates from a droplet containing initially 80% PG and 20% VG. Even for a large particle with a radius of 5 μm all the PG evaporates within a few seconds. In the experiment, PG evaporates much slower (~20 seconds) and does not evaporate completely either. These two differences between the simulation and experiment can only be explained by a change of the activity coefficient of PG because there is no source of PG vapor other than the droplet. Furthermore, the difference between the simulation and experiment is apparently independent of the water and nicotine content. Therefore, the reduction of the activity coefficient of PG must be attributed to the presence of VG
